# Impact of thermal fluctuations on phytoplankton: an experimental multi-trait analysis across species

**DOI:** 10.1093/plankt/fbaf021

**Published:** 2025-05-21

**Authors:** Osvaldo Tascón-Peña, Marco J Cabrerizo, María Pérez-Lorenzo, Emilio Marañón

**Affiliations:** Centro de Investigación Mariña da Universidade de Vigo, Illa de Toralla s/n, Vigo 36331, Spain; Department of Ecology and Animal Biology, Faculty of Marine Sciences, University of Vigo, Campus Lagoas Marcosende, Vigo 36310, Spain; Department of Ecology, Faculty of Sciences, University of Granada, Campus Fuentenueva s/n, Granada 18071, Spain; Centro de Investigación Mariña da Universidade de Vigo, Illa de Toralla s/n, Vigo 36331, Spain; Department of Ecology and Animal Biology, Faculty of Marine Sciences, University of Vigo, Campus Lagoas Marcosende, Vigo 36310, Spain; Centro de Investigación Mariña da Universidade de Vigo, Illa de Toralla s/n, Vigo 36331, Spain; Department of Ecology and Animal Biology, Faculty of Marine Sciences, University of Vigo, Campus Lagoas Marcosende, Vigo 36310, Spain

**Keywords:** environmental variability, elemental composition, growth rate, photosynthesis, photochemical performance, respiration, temperature

## Abstract

Thermal fluctuations affect the physiology and ecology of organisms. To date, most studies quantifying the effects of temperature on phytoplankton have used mean constant conditions, thus overlooking the role of short-term thermal fluctuations. Here, we use a multi-trait analysis to quantify how thermal regime (constant vs. fluctuation by ±3°C around mean temperature) alters the effect of temperature (18 vs. 22°C) on the growth, elemental composition, photosynthetic performance and metabolism of five phytoplankton species (*Emiliania huxleyi*, *Micromonas commoda*, *Skeletonema costatum*, *Synechococcus* sp. and *Thalassiossira rotula*) during exponential growth and stationary phases. Our results showed a high degree of inter-trait and inter-specific variability in the response to the temperature treatments. The carbon-based growth rates tended to be reduced by thermal fluctuations (by 20–29%), particularly under warming conditions. By contrast, thermal fluctuations increased the photosynthesis rates up to 25%, regardless of the growth phase. The carbon-to-nitrogen and carbon-to-chlorophyll *a* ratios, maximum photochemical yield of photosystem II and relative maximum electron transport rates did not show a clear response to interactions between thermal fluctuations and temperature. These results suggest that, when assessing phytoplankton responses to temperature, it is essential to consider both thermal fluctuations and multi-trait analysis.

## INTRODUCTION

Phytoplankton, the photosynthetic component of plankton, are responsible for half of the primary production on Earth (Field *et al.,* 1998) and constitute the main support of the food web in marine environments (Falkowski and Raven, 2007). This makes them a key group in biogeochemical cycles of carbon, nitrogen, phosphorus, silica and other elements (Falkowski *et al.,* 1998). Not all the organic matter generated by phytoplankton is remineralized in the euphotic layer; nearly 10 Pg C y^−1^ are exported to the ocean’s interior through a process called the biological pump (Bishop, 2009). Thus, due to the role of phytoplankton productivity and the biological pump in the regulation of atmospheric carbon dioxide (CO_2_) levels, it is imperative to understand how phytoplankton growth, stoichiometry and metabolism respond to anthropogenically- and climatically-driven environmental changes.

Temperature is one of the most relevant environmental drivers affecting phytoplankton, with a direct impact on their metabolism, growth and composition (Eppley, 1972; Raven and Geider, 1988; Finkel *et al.,* 2010). This driver has become even more relevant under the current global warming context, given ocean surface temperature has increased by 0.88°C in 2011–2022 relative to 1850–1900 and is projected to continue rising (IPCC, 2021). Additionally, global warming is increasing the frequency and intensity of marine heatwaves (Oliver *et al.,* 2018), which is subsequently increasing temperature variability of the surface ocean (Smale *et al.,* 2019). Despite increasing thermal variability in nature, most experimental studies that have addressed the effect of temperature on phytoplankton only have considered mean constant temperatures, thus neglecting the role of thermal fluctuations on phytoplankton ecology and physiology (Gunderson *et al.,* 2016; Kroeker *et al.,* 2020; Cabrerizo and Marañón, 2021). However, in nature, temperature changes occur on different time scales ranging from hours to months, depending on multiple environmental factors such as insolation, currents and weather (Bates *et al.,* 2018). According to Jensen’s inequality, the response of a given system to a mean constant driver is seldom equal to the mean response to variable ones (Jensen, 1906; Denny, 2017). In addition, thermally fluctuating regimes also increase the likelihood that supra-optimal temperatures are reached, thus potentially enhancing the risk of adverse effects on physiological performance (Vasseur *et al.,* 2014). Moreover, temperature fluctuations may impair the ability of cells to maintain metabolic homeostasis and balanced growth, due to the different thermal sensitivity and acclimation time scales of physiological sub-processes (Rehder *et al.,* 2023). Consequently, it is crucial to understand how temperature fluctuations affect phytoplankton functional traits in the context of global warming.

Previous studies suggest that thermal fluctuation can alter the responses of different phytoplankton traits. For instance, interactions between thermal fluctuations and warming potentially may reduce the biodiversity in freshwater ecosystems by selecting better adapted groups like cyanobacteria and chlorophytes (Rasconi *et al.,* 2017). Also, the growth of freshwater cyanobacteria is less reduced by fluctuation than that of their competitors (Zhang *et al.,* 2016). In marine ecosystems, fluctuating temperatures tend to increase diversity by creating opportunity windows to new species (Sarker *et al.,* 2020). Subsequent experiments using marine phytoplankton communities showed that interactions between warming and thermal fluctuations favored some chain-forming diatoms compared to other phytoplankton groups (Kling *et al.,* 2020). However, these effects could be minor compared with the effects on metabolism, where growth rate is reduced while carrying capacity is increased (Gerhard *et al.,* 2019). On the other hand, it has been observed that the negative effects of constant warming on phytoplankton photosynthesis and growth can be attenuated by thermal fluctuation (Cabrerizo *et al.,* 2021). In addition, the direction of the effects of thermal fluctuation can diverge among studies and species, which may be due to multiple factors like nutritional state, previous thermal history and the frequency, and duration and regularity of the thermal fluctuation (Fu *et al.,* 2022; Gill *et al.,* 2022; Kunze *et al.,* 2022). Although it is known that thermal fluctuations can influence phytoplankton responses, more across-species studies are needed to clarify the mechanisms involved, identify the most sensitive traits and establish general response patterns.

A relevant factor in the phytoplankton response to thermal fluctuations is the different sensitivity of species across different phylogenetic groups (Siegel *et al.,* 2023). For instance, it has been shown that a moderate warming (+4°C) increases biofilm formation, growth and evolutionary rates in *Thalassiosira pseudonana*, and that its combination with fluctuations in temperature enhance these three traits (Schaum *et al.,* 2018; Schaum, 2019). By contrast, *Emiliania huxleyi* growth, calcification and other physiological traits are negatively affected by thermal fluctuation, particularly under warmer temperatures (Wang *et al.,* 2019). Due to the large variability in the experimental designs followed to test the concurrent effects of warming and thermal fluctuations, it becomes necessary to conduct the same experiment with a variety of species to identify potential commonalities in eco-physiological responses. Applying the same experimental approach across different phylogenetic groups could also allow to establish a gradient of sensitivity of phytoplankton to the combined effects of warming and fluctuating temperature.

The effect of temperature upon phytoplankton growth and metabolism can be modulated by nutrient availability, as demonstrated by observational studies (Hayashida *et al.,* 2020) as well as experiments with phytoplankton cultures (Marañón *et al.,* 2018) and both coastal (O’Connor *et al.,* 2009) and open-ocean natural communities (Liu *et al.,* 2020; Fernández-González *et al.,* 2022). These studies have shown that nutrient limitation tends to reduce the effect of warming on multiple phytoplankton traits, including growth rates, light-harvesting capacity and photosynthetic carbon fixation. However, it is still unknown how the interaction between changes in temperature and nutrient availability plays out under a fluctuating thermal regime.

Here, we address two main questions to better understand the effects of warming in a thermally varying environment: (i) how do growth, photosynthetic performance, stoichiometry and metabolic rates respond to warming and thermal fluctuations? (ii) Does nutrient availability alter the sensitivity of phytoplankton to warming and thermal fluctuations? To determine the combined effects of thermal fluctuations and warming on phytoplankton, we exposed five phytoplankton species belonging to distinct phylogenetic groups to two temperature treatments (18 vs. 22°C) under two thermal regimes (constant vs. fluctuating) during contrasting growth phases (N-replete exponential growth phase and N-limiting stationary phase). We determined phytoplankton responses by measuring multiple functional traits, including growth rates, elemental composition, photochemical performance of photosystem II, and the rates of photosynthesis and respiration.

## MATERIALS AND METHODS

### Culture conditions and experimental design

We used five phytoplankton species belonging to different phylogenetic groups: *E. huxleyi* (Haptophyta), *Micromonas commoda* (Chlorophyta), *Synechococcus* PCC7002 (Cyanobacteria), *Skeletonema costatum* and *Thalassiosira rotula* (Bacillariophyta). Cultures were grown in f/4 medium, modified to have a nitrogen to phosphorus molar ratio of 10 to favor nitrogen limitation at the end of the exponential growth phase (Marañón *et al.,* 2013). Although there are exceptions, phytoplankton N:P tends to be well above 10 (Garcia *et al.,* 2018) which means that N is likely to have been the limiting nutrient at the onset of the stationary phase in most of our cultures. We maintained the cultures under four different temperature treatments, two of which were constant (18°C and 22°C) and two of which were fluctuating (±3°C around 18 and 22°C). The four temperature treatments were: control-constant (C; 18°C), heated-constant (H; 22°C), control-fluctuating (CF) and heated-fluctuating (HF). Acclimation was conducted in semi-continuous cultures (one per treatment) maintained in 1-L Erlenmeyer flasks, with a dilution rate of 20% every two days. All species were acclimated for at least two weeks to both temperature and thermal regimes (constant or fluctuating) before the start of the experiment. We consider that we were working with a fully acclimated phenotype since, during this period, populations went through multiple generations: 9.3 for *E. huxleyi*, 10.4 for *M. commoda*, 13.6 for *S*. *costatum*, 8.2 for *Synechococcus* and 7.8 for *T*. *rotula*. During this period, the cultures were maintained in growth chambers under a 12:12 Light:Darkness photoperiod, receiving 250 μmol photon m^−2^ s^−1^ of photosynthetically active radiation (PAR) provided by LED lights (HomePluss® 119—Garcasso Leds, Spain). The temperature inside the growth chambers was continuously monitored by TinyTag data loggers (TinyTag Aquatic 2, UK; chambers accuracy ±0.5°C), and the thermal fluctuations (i.e. changes above/below the mean temperature) had a 48 h cycle, with the temperature changing in the middle of the light phase. Fluctuating temperature treatments changed regularly every 24 h with a 5-h transition time (increasing or decreasing temperature) at a rate of 0.6°C h^−1^ between 19-h constant temperature periods. Therefore, during the two-week acclimation period, cultures underwent seven complete fluctuation cycles. During the batch period, they experienced at least two to five complete fluctuation cycles, depending on the species considered. We used 18°C as our control temperature because it is the temperature at which the strains used had been maintained in our phytoplankton culture collection over the last decade. According to previous studies (Krawiec, 1982; Thomas *et al.,* 2016) showed that the thermal optima of the species used were as follows: 21°C (±2.4 SD) for *E. huxleyi*, 21°C (±1.1 SD) for *M*. *commoda*, 21.7°C (±4.4 SD) for *S*. *costatum*, 29°C (±1.4 SD) for *Synechococcus* sp. and 21.9°C (±3.6 SD) for *T*. *rotula*. Therefore, temperatures in the control-fluctuating regime were mostly below the thermal optimum, constant heated temperature was slightly above the expected thermal optimum (except for *Synechococcus*) and heated-fluctuating temperatures exceeded the expected thermal optimum during 24 h (except for *Synechococcus*).

Once acclimated, triplicate 1-L cultures were exposed to the four temperature treatments described above to investigate the effects of warming and thermal fluctuations during the exponential growth phase (under nutrient-replete conditions) and once populations had reached the stationary phase (after the onset of nutrient limitation). All cultures were gently stirred several times during the light period to prevent cells from settling and were randomly distributed inside the chamber to avoid any position effect. Additionally, for each species, we sampled all treatments (C, H, CF and HF) on the same experimental day to ensure comparability of responses. However, populations may not always have been at the same point in the growth curve (see Results).

### Biomass and standing stocks

To determine the concentration of chlorophyll *a* (Chl *a*), particulate organic carbon (POC) and particulate organic nitrogen (PON), samples of 20 mL were collected from each flask, filtered thorough pre-combusted (5 h, 500°C) GF/F filters (Whatman Inc., United Kingdom) and frozen at −20°C until analysis. Chl *a* samples were extracted in 90% acetone at 4°C in darkness overnight and measured with a Trilogy fluorometer (Turner Designs, San Jose, CA, USA). Samples for POC and PON determinations were thawed, dried at room temperature for three days and measured using a Flash EA1112 elemental analyzer (Thermo Scientific, Walthman, MA, USA).

### *In vivo* fluorescence and photosystem II activity

*In vivo* Chl *a* fluorescence was measured with a pulse amplitude modulated (PAM) fluorometer (Phyto-PAM II Compact version, Walz, Effeltrich, Germany) equipped with an optical unit (ED101-US). Samples of 15 mL were collected daily at 12:00 a.m., placed in opaque Falcon tubes (Corning®, USA) and acclimated to darkness for 15 min prior to being measured. Previously to being used in experimentation, and to have absolute Chl *a* concentrations, we calibrated the device using *ad hoc* reference spectra generated from extracted Chl *a* data of the target species. From the same samples, we measured the maximal photochemical quantum yield of photosystem II (Fv/Fm) using the equation by Genty *et al.* (1989) as:


$$ \mathrm{Fv}/\mathrm{Fm}=\left({\mathrm{F}}_{\mathrm{m}}\hbox{--} {\mathrm{F}}_{\mathrm{o}}\right)/{\mathrm{F}}_{\mathrm{m}} $$

where F_m_ is the maximum fluorescence induced by a saturating light pulse (5000 μmol photons m^−2^ s^−1^) and F_o_ the minimal fluorescence induced by a dim pulse of actinic light (10 μmol photons m^−2^ s^−1^), too weak to induce reaction center closure. The samples were also used to perform rapid light curves (RLCs) by exposing the dark-adapted cells to 12 consecutive increasing actinic light pulses (1–1 132 μmol photons m^−2^ s^−1^) with each pulse lasting 10 s. We calculated the maximum relative electron transport rate (rETR_max_) by fitting the rETR rate vs. irradiance data using the model by Platt *et al.* (1980).

### Photosynthesis and respiration

Samples of 35 mL in volume were taken from each culture flask, gently transferred to narrow-neck borosilicate bottles (VWR®, Romania, EU) avoiding the formation of bubbles, and incubated for 4 h in the corresponding temperature and thermal regime treatment where they came from, under the same light conditions (for the determination of net photosynthesis) or under darkness (for respiration). Net photosynthesis and respiration rates were determined by the Winkler technique using a potentiometric end-point. Both rates were converted into carbon units assuming an O_2_ to CO_2_ ratio of 1.4 (mol:mol; Laws, 1991), and then normalized by POC concentration to obtain C-specific rates.

### Data treatment and statistical analysis

POC-based specific growth rates (*μ*) were calculated as:


$$ \mu =\ln \left({\mathrm{N}}_1-{\mathrm{N}}_0\right)/\left({\mathrm{T}}_1-{\mathrm{T}}_0\right) $$

where N_0_ and N_1_ are the POC concentrations measured at the start of the experiment (time = T_0_) and during the exponential phase (time = T_1_), respectively. *In vivo* Chl *a* fluorescence-based growth rate was calculated as the slope of the linear regression (R^2^ > 0.97 for all treatments/species) of the natural logarithm of Chl *a* vs. time during the exponential phase.

A two-way analysis of the variance was used to test significant differences between treatments in growth rates, C:N, C:Chl *a*, photosynthesis, respiration, Fv/Fm and rETR_max_. All species and growth phases were analyzed separately. Assumptions of normality (by Shapiro–Wilk’s test) and homoscedasticity (by Levene’s test) of the data were checked before using analyses of variance (ANOVAs) with the package “car” in R statistical software (v. 4.2.3). When data did not meet ANOVA assumptions, they were transformed using a box-cox transformation with “MASS” R package. When the interaction between temperature and the thermal regime was significant, a Turkey *post hoc* test was used to assess differences within treatments. For those response variables that did not satisfy ANOVA assumptions after being transformed, we used a non-parametric Kruskal–Wallis test followed by a Dunn *post hoc* test using “PMCMRplus” R package.

The effect of heat, fluctuating temperature and its combination (heat-fluctuating) was quantified as a relative change (RC_c_), in percentage, between each treatment and the control-constant treatment:


$$ {\mathrm{RC}}_{\mathrm{c}}=100\times \left(\mathrm{Treatment}-{\mathrm{Control}}_{\mathrm{c}\mathrm{onstant}}\right)/{\mathrm{Control}}_{\mathrm{c}\mathrm{onstant}} $$

where Treatment represents the value of a given variable measured in the heated-constant, control-fluctuating or heated-fluctuating treatments, and Control_constant_ is the value measured in the control-constant temperature treatment. We also calculated the effect of fluctuating temperature under heated conditions as a relative change (RC_h_):


$$ {\mathrm{RC}}_{\mathrm{h}}=100\times \left({\mathrm{Heated}}_{\mathrm{fluctuating}}-{\mathrm{Heated}}_{\mathrm{constant}}\right)/{\mathrm{Heated}}_{\mathrm{constant}} $$

where Heated_fluctuating_ and Heated_constant_ are the values of a given variable measured in the heated-fluctuating and the heated-constant treatments, respectively.

To assess the extent to which measured traits diverged among different species, temperature treatments and regimes, and the growth phase considered, a principal component analysis (PCA) was conducted, as proposed by Walworth *et al.* (2021). Specifically, a trait-scape was created using eight independent traits in the exponential phase, and six in the stationary one. The contributions of each trait to the PC axes were extracted using the “factoextra” package in R. We replaced 14 absents data in our dataset with the mean of the 2 correspondent replicates data. Normalization was performed using “scale” function in R to ensure equal treatment of the traits.

## RESULTS

### Temporal response of chlorophyll *a*

**Fig. 1:**
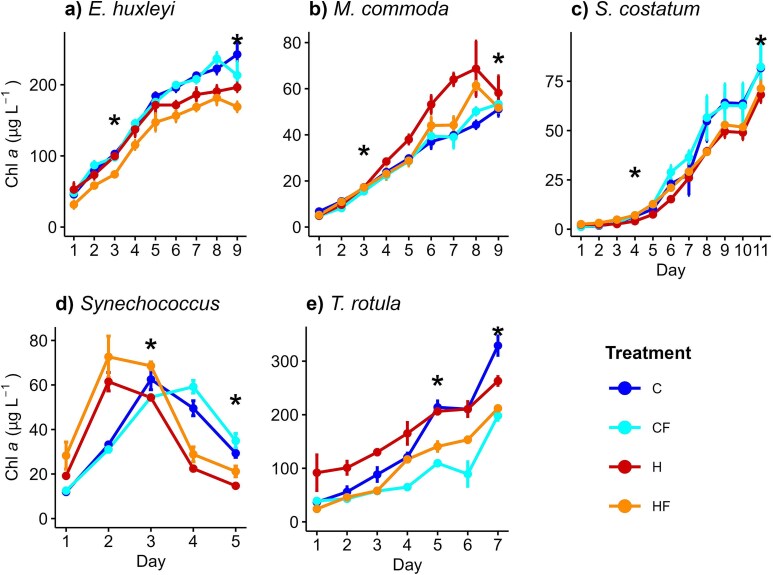
Temporal response of mean (±SD) fluorescence-based Chl *a* concentration in (**a**) *E*. *huxleyi*, (**b**) *M*. *commoda*, (**c**) *S*. *costatum*, (**d**) *Synechococcus* and (**e**) *T. rotula* exposed to different temperature treatments: control-constant (18°C; C), control-fluctuating (18 ± 3°C; CF), heated-constant (22°C; H) and heated-fluctuating (22 ± 3°C; HF). Asterisks indicate sampling days.

The temporal response of Chl *a* concentration over time revealed that warming and thermal fluctuations had a species-specific effect. The increase in culture Chl *a* concentration registered in *E. huxleyi* and *S*. *costatum* was inhibited by H and HF treatments compared to C conditions (Fig. 1a and e), whereas these treatments stimulated Chl *a* concentration in *M. commoda* (Fig. 1d). In *Synechococcus*, we found that the effect of H and HF treatments accelerated the increase of Chl *a* concentration, which reached the highest value in the HF treatment (Fig. 1c). Finally, in *T*. *rotula*, we found that the accumulation of Chl *a* was lower under fluctuating temperatures, CF and HF, compared to their corresponding constant temperature, C and H, over the experimental period (Fig. 1b).

### Responses of growth and elemental composition during the exponential growth phase

**Fig. 2:**
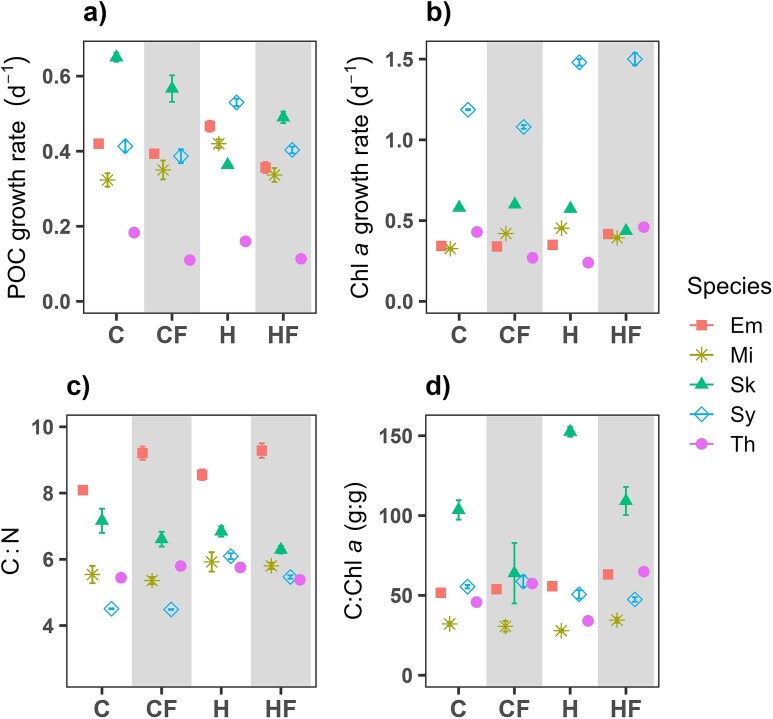
Mean (±SD) (**a**) POC-based growth rates, (**b**) Chl *a*-based growth rates, (**c**) carbon to nitrogen ratio (C:N) and (**d**) carbon to Chl *a* ratio (C:Chl *a*) measured during the exponential growth phase in *E. huxleyi* (Em, square), *M*. *commoda* (Mi, asterisk), *S*. *costatum* (Sk, triangle), *Synechococcus* (Sy, diamond) and *T*. *rotula* (Th, circle) exposed to different temperature treatments: control-constant (18°C; C), controlfluctuating (18 ± 3°C; CF), heated-constant (22°C; H) and heated-fluctuating (22 ± 3°C; HF). Standard deviation bars do not appear when they are smaller than the symbol size.

**Fig. 3:**
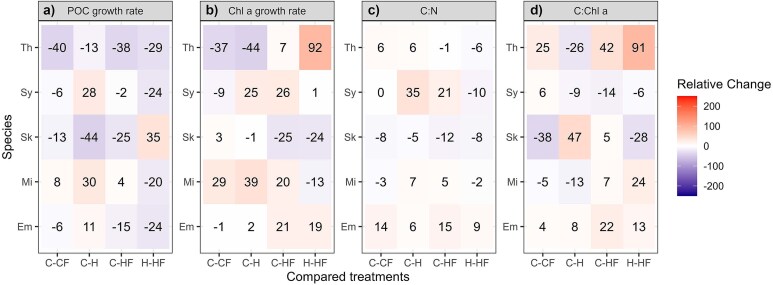
Mean relative change (%) of POC-based growth rate, Chl *a*-based growth rate, C:N and carbon to Chl *a* (C:Chl *a*) ratio measured during the exponential growth phase in *E*. *huxleyi* (Em), *M*. *commoda* (Mi), *S*. *costatum* (Sk), *Synechococcus* (Sy) and *T*. *rotula* (Th). In the first three columns, relative change is shown for the comparisons between each treatment with respect to the control (C–CF, C–H and CHF) and in the last column, the relative change is shown for the heated-fluctuating versus heated-constant treatments comparison (H–HF).

POC-based growth rates ranged between 0.32 and 0.65 d^−1^, except for *T*. *rotula* where they were around 0.1–0.2 d^−1^ (Fig. 2a). For *E. huxleyi*, *M*. *commoda* and *Synechococcus*, the highest growth rates were obtained under H treatment, whereas in *S*. *costatum* and *T*. *rotula* they were measured under C conditions (Fig. 2a). Fluctuation, as a single driver and compared to C conditions, did not have a significant effect on POC-growth rate, except for *S*. *costatum* in which it was marginally inhibitory (RC_c_ = −13%; Tukey *post hoc* test, *P* = 0.07) and *T*. *rotula* where it was significantly negative (RC_c_ = −40%; Tukey *post hoc* test, *P* < 0.001; Fig. 3a). When we compared the HF with H treatments, fluctuation reduced the POC-based growth rate in *E. huxleyi*, *M*. *commoda*, *Synechococcus* and *T*. *rotula* by between −20 and −29% (Tukey *post hoc* test, *P* < 0.05; Fig. 3a). In *E. huxleyi*, *M*. *commoda* and *Synechococcus*, constant warming had a positive effect on POC-based growth relative to C, but the effect of heated-fluctuating conditions, relative to the control, was neutral or negative (Fig. 3a). However, due to the choice of sampling times, the growth rate of *Synechococcus* may have been underestimated.

Considering Chl *a*-based growth rates, *Synechococcus* was the species that exhibited the highest rates, particularly under H and HF treatments (1.5 d^−1^; Fig. 2b). In the other species, these rates showed values ≤ 0.6 d^−1^ regardless of the temperature treatment considered (Dunn *post hoc* test, *P* > 0.1 in all cases). In general, compared with C, this trait was not significantly affected by a CF temperature regime except for *T*. *rotula*, which showed a reduction of 37% (Tukey *post hoc* test, *P* = 0.004), and *M. commoda* which had an increase of 29% (Tukey *post hoc* test, *P* < 0.001; Fig. 3b). In contrast, the relative change between H and HF was 19% in *E. huxleyi* and 91% in *T*. *rotula*, whereas RC values of −13% and − 24% were measured for *M*. *commoda* and *S*. *costatum.* Additionally, *M*. *commoda* and *Synechococcus* Chl *a* based growth rate was increased by H and HF treatments compared to the C treatment by 20% to 39% (Fig. 3b).

C:N ratios varied between 4.2 and 9.3 and did not show a clear pattern of response to the individual or combined effect of the drivers tested (Figs 2c and3c; Dunn or Tukey *post hoc* tests, *P* > 0.05). The C:Chl *a* ratio showed values < 50 in all species except for *S*. *costatum,* where they reached values up to 150 (Fig. 2d). Fluctuating temperature and warming exerted a significant but opposite effect on the C:Chl *a* ratio of *S*. *costatum* and *T*. *rotula*. Whereas CF temperature stimulated by 25% this ratio in *T*. *rotula* and reduced it by 38% in *S*. *costatum*, the H treatment reduced it by 26% and stimulated it by 47% in the above-mentioned species. The HF treatment, compared to H, increased the C:Chl *a* between 13 and 91% in *E*. *huxley*, *M*. *commoda* and *T*. *rotula*, but in *S*. *costatum* it caused a reduction of 28% (Tukey *post hoc* test, *P* < 0.05) in *S*. *costatum* (Fig. 3d).

### Responses of metabolic rates during the exponential growth phase

**Fig. 4:**
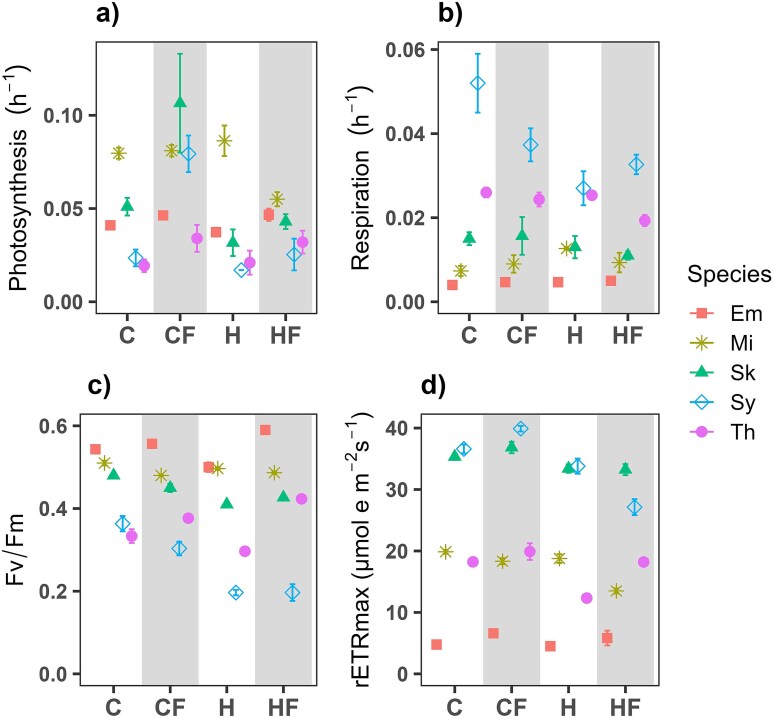
Mean (±SD) (**a**) carbon-specific photosynthesis rate, (**b**) carbon-specific respiration rate, (**c**) maximum photochemical yield of photosystem II (Fv/Fm) and (**d**) relative maximum electron transport rate (rETRmax) measured during the exponential growth phase in *E. huxleyi* (Em, square), *M*. *commoda* (Mi, asterisk), *S*. *costatum* (Sk, triangle), *Synechococcus* (Sy, diamond) and *T*. *rotula* (Th, circle) under different treatments: control-constant (18°C; C), control-fluctuating (18 ± 3°C; CF), heated-constant (22°C; H) and heated-fluctuating (22 ± 3°C; HF). Standard deviation bars do not appear when they are smaller than the symbol size.

**Fig. 5:**
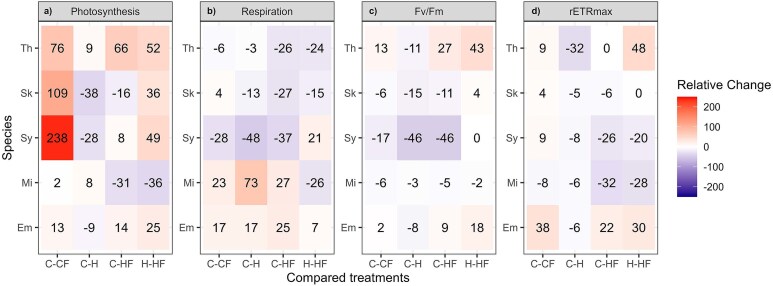
Mean relative change (%) of carbon-specific photosynthesis and respiration, maximum photochemical yield of photosystem II (Fv/Fm) and rETRmax measured during the exponential growth phase in *E*. *huxleyi* (Em), *M*. *commoda* (Mi), *S*. *costatum* (Sk), *Synechococcus* (Sy) and *T*. *rotula* (Th). In the first three columns, relative change is shown for the comparisons between each treatment with respect to the control (C–CF, C–H and C–HF) and in the last column, the relative change is shown for the heated-fluctuating versus heated-constant treatments comparison (H–HF).

Carbon-specific photosynthesis rates varied between 0.01–0.06 h^−1^ with some exceptions like *M*. *commoda* under C, CF and H (from 0.8 to 0.9 h^−1^), *S*. *costatum* (0.11 h^−1^) under the CF treatment, and *Synechococcus* (0.08 h^−1^; Fig. 4a). The comparison between H and C did not show a clear common response in *E. huxleyi*, *M*. *commoda* and *T*. *rotula*. The relative change between CF and C revealed an increment under the fluctuation regime higher than 10% in *E*. *huxley*, *S*. *costatum*, *Synechococcus* and *T*. *rotula*. This is consistent with the relative change between HF and H, where fluctuation increased photosynthesis by more than 25% in the same species. *M*. *commoda* seems to be the exception, with photosynthesis affected negatively only under HF treatment. Carbon-specific respiration rates ranged between ~ 0.01 and 0.06 h^−1^, were lowest in *E. huxleyi* and highest in *Synechococcus* (Fig. 4b). In *E. huxleyi* and *M*. *commoda*, CF, H and HF increased respiration more than 17% relative to C, whereas in *S*. *costatum*, *Synechococcus* and *T*. *rotula* respiration in HF decreased more than 26% relative to C. Moreover, the HF treatment compared to H led to a reduction in respiration between 15% and 26% in *M*. *commoda*, *S*. *costatum* and *T*. *rotula* (Fig. 5b).

Fv/Fm ranged between 0.2 (*Synechococcus*) and 0.6 (*E. huxleyi*; Fig. 4c). A reduction of Fv/Fm in the H and HF treatments, relative to C treatment, was found in *M. commoda*, *S*. *costatum* and *Synechococcus*, the difference being significant in most of the cases (Tukey *post hoc* test, *P* < 0.05; Fig. 5c). Similarly to what was observed for Fv/Fm, rETR_max_ rates showed a considerable range of variation between species (< 10 to 50 μmol e^−^ m^−2^ s^−1^; Fig. 4d). In all species, rETR_max_ rates were reduced in the H treatment, relative to C. Fluctuation exerted a slight positive effect on rETR_max_ for almost all the species tested when compared with C, but a positive, negative or neutral effect when compared to H (Fig. 5d).

### Responses of elemental composition and metabolic rates during the stationary phase

**Fig. 6:**
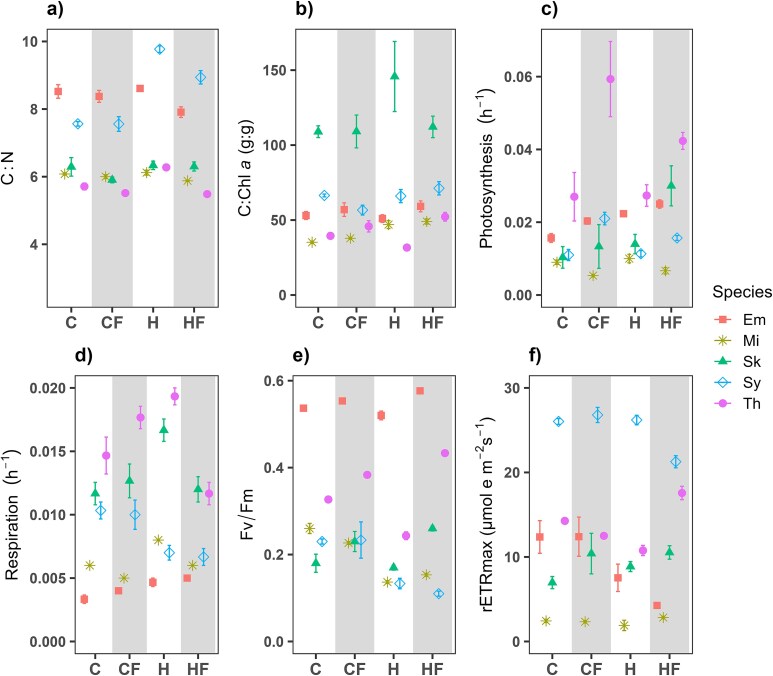
Mean (±SD) (**a**) carbon to nitrogen (C:N ratio), (**b**) carbon to Chl *a* (C:Chl a), (**c**) carbon-specific photosynthesis, (**d**) carbon-specific respiration, (**e**) maximum photochemical yield of photosystem II (Fv/Fm) and (**f**) rETRmax measured during the stationary phase in *E. huxleyi* (Em, blue square), *M*. *commoda* (Mi, asterisk), *S*. *costatum* (Sk, triangle), *Synechococcus* (Sy, diamond) and *T*. *rotula* (Th, circle) under the different treatments: control (18°C; C), control with fluctuation (18 ± 3°C; CF), heated (22°C; H) and heated with fluctuation (22 ± 3°C; HF).

**Fig. 7:**
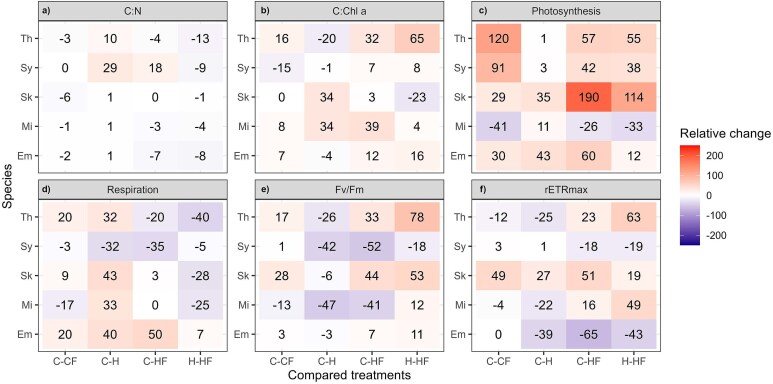
Mean relative change (%) of carbon to nitrogen (C:N) ratio, carbon to Chl *a* (C:Chl *a*) ratio, carbon-specific photosynthesis and respiration, maximum photochemical yield of photosystem II (Fv/Fm) and rETRmax measured during the stationary growth phase in *E*. *huxleyi* (Em), *M*. *commoda* (Mi), *S*. *costatum* (Sk), *Synechococcus* (Sy) and *T*. *rotula* (Th). In the first three columns, relative change is shown for the comparisons between each treatment with respect to the control (C-CF, C–H and C–HF) and in the last column, the relative change is shown for the heated-fluctuating versus heated-constant treatments comparison (H–HF).

In most species and treatments, we observed an increase of C:N and C:Chl *a* ratios in the stationary phase with respect to the exponential growth phase (Fig. 2c and d and Fig. 6a and b). In general, C:N did not show any clear response to the drivers tested when compared to C conditions (Fig. 7a). Similarly to what was observed in the exponential phase, *S*. *costatum* had the highest C:Chl *a* ratio of all species tested (Fig. 6b). Again, this trait exhibited higher variability than C:N ratio under the temperature treatments. While the H treatment compared to the C did not show a common response, HF had a positive relative change respect to C in all species. The HF treatment, compared to H, resulted in an increase in C:Chl *a* in all species except for *S*. *costatum* (Fig. 7b).

Carbon-specific photosynthesis and respiration showed, as expected, lower rates during the stationary phase than during the exponential growth phase, except for *T*. *rotula* photosynthesis (Fig. 6c and d). During the stationary phase, photosynthesis rates were typically lower than 0.05 h^−1^ (Fig. 6c). Constant heating compared with C increased the photosynthesis by between 11 and 43% in *E. huxleyi*, *M*. *commoda* and *S*. *costatum*. We observed a positive effect of fluctuation on photosynthesis when assessing the relative change between C and CF as well as between H and HF in all the species except for *M*. *commoda* (Fig. 7c). On the other hand, respiration was not significantly affected by CF and H temperatures, with the exception of the two diatoms where HF temperature reduced the respiration from 0.016 d^−1^ and 0.019 d^−1^ to 0.012 d^−1^ and 0.011 d^−1^ (Fig. 6d; Tukey *post hoc* test, *P* ≤ 0.05). H increased respiration, relative to C, by 32–43% (Fig. 7d), with the difference being significant in *E. huxleyi*, *S*. *costatum* and *T*. *rotula* (Tukey *post hoc* test, *P* < 0.05).

Maximum photochemical yield (Fv/Fm) showed a high variability of responses under the assessed conditions. In general *E. huxleyi* and *T*. *rotula* showed higher values than the rest of the species (Fig. 6e). In *E. huxleyi*, *S*. *costatum* and *T*. *rotula* fluctuation slightly increased the Fv/Fm when compared to C temperature, and this increase was slightly higher when comparing H and HF (Fig. 7e). HF treatment had a stronger, positive effect than H respect to the control temperatures, with the difference between HF and C being significant in all cases (Tukey *post hoc* test, *P* < 0.05). The responses of rETR_max_ were mostly in agreement with those of Fv/Fm, with most of the significant differences (Tukey *post hoc* test, *P* < 0.05) being found between the control and the HF in *E. huxleyi*, *S*. *costatum* and *T*. *rotula* (Figs 6f and7f).

### Interspecific differences in multi-traits variability

**Fig. 8:**
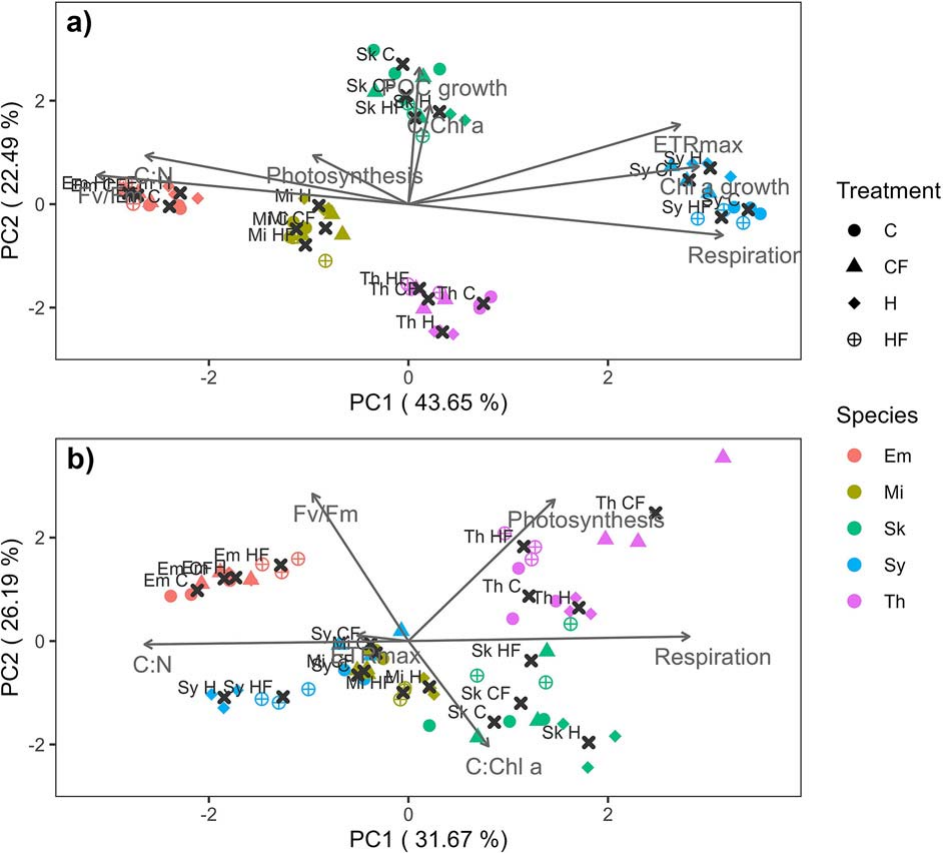
PCA of (**a**) eight and (**b**) six traits measured during the exponential and stationary growth phases, respectively. Color indicates the species: *E*. *huxleyi* (Em), *M*. *commoda* (Mi), *S*. *costatum* (Sk), *Synechococcus* (Sy) and *T*. *rotula* (Th). The shape indicates the treatment: control (dot, C), control with fluctuation (triangle; CF), heated (diamond; H) and heated with fluctuation (cross with circle; HF). Centroids are indicated with a black cross.

The PCA showed that the first two orthogonal axes captured more than 58% and 66% of the trait variance for the stationary and exponential growth phases, respectively. We observed four (stationary phase) and five (exponential phase) distinct clusters that clearly separated the five species (Fig. 8), hence the main factor shaping the response in our experiment is the species factor. In the exponential growth phase, the variables that contributed the most to PC1 were respiration (22.8%), Fv/Fm (22.4%) and Chl *a*-based growth rate (19.5%) whereas for the PC2 were POC-based growth rate (43.6%), C:Chl *a* ratio (23%) and the rETR_max_ (14.8%). In the stationary phase, *Synechococcus* and *M. commoda* were grouped together, and the response variables that most contributed to the explained variance were respiration (42%), C:N ratio (37%) and photosynthesis (11.4%) for the PC1, while for PC2 were Fv/Fm (41%), photosynthesis (37.9%) and the C:Chl *a* (20.9%; Fig. 8; Table S1).

Comparing both PCA plots, we observed that *S*. *costatum* is associated with the direction of the C:Chl a trait, while *E. huxleyi* and *M. commoda* are associated with the C:N ratio, and *Synechococcus* aligns with rETRmax, which reduces its contribution during the stationary phase. Additionally, interspecific separation decreases in the stationary phase, whereas intraspecific variability increases. This indicates that phenotypic variability is greater between species, particularly under nutrient-limiting conditions, as evidenced by the increased dispersion of species along the PC2 axis (Fig. 8). Specifically, PC2 coordinates ranged from −2.51 to 2.61 when PC1 > 0 compared to −1.54 to 2.98 when PC1 < 0 under nutrient-replete conditions. In contrast, under nutrient-limiting conditions, PC2 coordinates ranged from −2.44 to 3.54 and − 1.29 to 1.5, respectively. This pattern suggests that as metabolism accelerates, other traits tend to exhibit greater variability.

## DISCUSSION

We provide experimental evidence on the effect of thermal fluctuations and increased temperatures on multiple-traits of five phytoplankton species under two different nutritional states. As predicted by Jensen’s inequality, our results show that fluctuating temperatures, compared to control ones, either stimulated or inhibited the traits tested. These findings are in line with the proposal by Wolf *et al.* (2024) that temperature effects on phytoplankton can be positive or negative depending on whether exposure takes place uniformly or oscillates above or below mean trends. Thus, responses to short-term thermal variability proved less intuitive than constant temperatures as they are driven by a complex interplay between cool and warm phases. Although we found a clear effect of thermal fluctuations on all species tested, these responses were strongly species-specific considering the entire range of traits assessed. Nevertheless, some common trends were identified, in particular for growth rate and photosynthesis.

Several reasons may have caused the differences observed between species in the response to temperature changes. The cell size (diameter) of the species tested ranged between 1 μm in *Synechococcus* and > 60 μm in *T*. *rotula*, which likely affects their physiology since cell size correlates with multiple traits including cellular stoichiometry, growth and metabolic rates (Finkel, 2001; Marañón, 2015). Recent findings by Barton *et al.* (2023) indicate varying levels of thermal adaptation among phytoplankton species exposed to supra-optimal temperatures. For instance, smaller species such as *Synechococcus* and *Ostreococcus* demonstrated improved fitness and/or thermal tolerance, allowing them to better adapt to warming. In contrast, larger species like *Phaeodactylum tricornutum* failed to adapt. Generally, smaller phytoplankton tend to dominate in warmer seas, as they are better equipped to cope with oligotrophic conditions compared to larger cells (Marañón, 2015; Hillebrand *et al*., 2022). Given the relationship between mean *in situ* temperature and thermal traits (Chen, 2015), small-sized species with higher optimal temperatures for growth may outperform larger species under warming conditions. The employed species also had different growth rates, and it has been previously seen that fast-growing species tend to show wider thermal tolerance (Chen, 2015). This author hypothesized that rapid growth might be explained by higher concentrations of rate-limiting enzymes, such as RubisCO, allowing positive growth to be sustained even when these enzymes are partially denatured at extreme temperatures. Therefore, generation time could also condition the phytoplankton responses. In this regard, Walworth *et al.* (2020) have shown that for the same physical dynamics (i.e. fluctuating temperatures), a slower growing population would experience a more variable environment while a faster growing one would experience a more stable one. In fact, Jackson *et al.* (2021) have proposed that it is more meaningful to think in terms of the time scales at which organisms operate rather than absolute time *per se* when we address the impacts of global change drivers. This view has been recently supported by the finding that the overall physiological behavior in response to temperature is the result of the individual responses of each trait i.e. while two different species may grow at the same rate, their metabolisms can be operating at different states (Fu *et al.,* 2022). Therefore, the fact that our species had different growth rates, which ultimately indicate that they operated at different time scales (and had different generation times), may have influenced the responses detected.

Another important factor contributing to the observed variability could be the differential temperature sensitivities of the traits (metabolism, physiology and standing stocks) measured. For example, previous results have showed that the thermal optimum for primary productivity or Chl *a* content in *T. pseudonana* is higher than that for its growth rate (Baker *et al.,* 2016), that the thermal optimum for maximum quantum yield is lower than that for growth in *Chatonella subsalsa* (Vidyarathna *et al.,* 2020) or higher for the ETR rates than for Fv/Fm in different Arctic diatoms (Rehder *et al.,* 2024).

We used a 2D multivariate analysis to search for common trends, integrating multiple traits of the phenotypic phytoplankton response to thermal fluctuation (Walworth *et al.,* 2021; Argyle *et al.,* 2021a). We found a considerable inter-trait and interspecific variability in the responses to fluctuating temperatures and warming, both as single and combined drivers. The integrated trait-scapes also evidenced that the interspecific variability exceeded the variations caused by temperature and the thermal regime regardless the phase of the growth considered. These findings are consistent with those reported by Ye *et al.* (2023) in dinoflagellates and diatoms, although these authors only considered the individual effects of temperature (without considering fluctuations). During the exponential phase, interspecific variability is higher compared to the stationary phase. This suggests that traits tend to become more variable under accelerated metabolic activity. Conversely, in the stationary phase, intraspecific differences increase, and interspecific distances decrease. This indicates that under nutrient limitation, species-specific factors variability tends to diminish due to reduced metabolic activity. While the study of functional traits has gained attention in phytopankton ecology in recent decades (Litchman and Klausmeier, 2008; Ayata *et al.,* 2014; Argyle *et al.,* 2021b), only a few studies have addressed the integrated trait variations between species in a global change context. We stress the need of considering multi-trait scapes to better predict the impacts of global change drivers on phytoplankton.

An increase in temperature may stimulate or depress the growth rate (or any other trait), depending on whether it takes place in the region below or above the optimal temperature. The trait response increases its complexity when evaluated under a thermally fluctuating environment. Diurnal or slower fluctuating frequencies tend to reduce the phytoplankton performance whereas faster ones increase it, when compared to constant temperature conditions (Kunze *et al.,* 2022). Also, it has been shown that thermal fluctuations can reduce the optimal temperature for growth in *Tetraselmis tetrahele* and *E. huxleyi* (Bernhardt *et al.,* 2018; Wang *et al.,* 2019). Previous studies that used our species showed that their thermal optimum was around 20–22°C except for our strain of *Synechococcus*, whose optimal temperature for growth is 29°C (Krawiec, 1982; Thomas *et al.,* 2016). This higher thermal optimum of *Synechococcus* likely explain the higher chlorophyll-based growth rates observed in the H and HF treatments compared to the C and CF treatments. The ongoing warming of the surface ocean seems to increase the thermal optimum, as observed by Hattich *et al.* (2024) in Baltic Sea *Skeletonema marinoi* populations, which have increased its thermal optimum by 1°C over the last 60 years. In the case of our experiment, however, given all the species tested had been maintained at 18°C for a decade prior to being used in experimentation, it is unlikely that their thermal optimum had changed markedly.

Our results showed that, in most of the cases, the thermal fluctuation compared to constant temperature, reduced POC-based growth rate, with this reduction being higher at the warmer temperature. The mechanism underpinning such response is likely that in the CF treatment the temperature fluctuated between 15 and 21°C, hence remaining below the hypothetical thermal optimum of the species, while HF treatment fluctuated between 19 and 25°C, exceeding the hypothetical thermal optimum in four of the species tested. The negative effect of thermal fluctuation on growth rates has been previously reported in *Chlorella pyrenoidosa*, *Cyclotella meneghiniana* and *E. huxleyi* (Zhang *et al.,* 2016; Wang *et al.,* 2019; Zhang *et al.,* 2019). By contrast, a constant 4°C warming increased the POC-based growth rate in *E. huxleyi*, *M. commoda* and Sy, which likely reflects that 22°C is closer to the thermal optimum than 18°C. The patterns observed for POC-based growth differ somewhat from those obtained with Chl *a*-based growth rates. These differences could be explained by the variable time resolutions of the methodologies used to quantify both response variables and the tendency of cells to become richer in Chl *a* during the exponential growth phase (Fernández-González and Marañón, 2021; Kruskopf and Flynn, 2006).

Photosynthesis has a lower thermal dependence than respiration (Padfield *et al.,* 2016; Marañón *et al.,* 2018; Barton *et al.,* 2020). Thus, an increase in temperature tends to stimulate respiration more than photosynthesis, leading to a reduction in the carbon available for growth. Therefore, the balance between these two processes is a crucial aspect of the phytoplankton response to temperature changes (Allison, 2014). Our results showed that, compared with the corresponding constant mean temperature, the thermal fluctuation treatment resulted in higher photosynthesis rates (except in *M*. *commoda*) during both the exponential growth phase and the stationary phase. An enhancement of photosynthesis was previously reported in 48-h fluctuations of 2.5°C around 25.5°C in *E. huxleyi* (Wang *et al.,* 2019). The different response of photosynthesis and growth to thermal fluctuations may have resulted, at least in part, from the fact that the first process was quantified over short-term scales (4 h) whereas the second one was calculated from data collected over several days. Thermal fluctuation in the upper thermal limit can increase metabolic rates to tolerate periodic exposure to temperature stress, although this metabolic enhancement may not necessarily be associated with a stimulation of growth rates (Fu *et al.,* 2022). Therefore, photosynthesis can be increased as a metabolic response to thermal fluctuations (Padfield *et al.,* 2016; Wang *et al.,* 2019). As a complementary method to assess the photosynthetic performance, we used two photophysiological metrics, Fv/Fm and rETR_max_. The lack of the effect of thermal fluctuations on both variables is in line with a previous work that showed no clear response of Fv/Fm in the diatom *C. meneghiniana* and the green algae *C. pyrenoidosa* (Zhang *et al.,* 2019). By contrast, Fv/Fm seems to be reduced by constant heating respect to control, as well as by nutrients limitation experienced during the stationary growth phase. These findings are consistent with the negative effect of warming previously described by Mai *et al.* (2021) in natural phytoplankton communities, and with reductions found in Fv/Fm for fourteen marine phytoplankton species exposed to nitrogen or phosphorus limitation (Tan *et al.,* 2019). The latter authors proposed that such response variable can be used as a proxy to determine nutrients limitation on phytoplankton.

As expected, we observed reductions in photosynthesis, respiration, rETR_max_ rates and Fv/Fm under nutrient deficiency during the stationary phase compared with those measured during the exponential growth phase. We also expected an attenuation of temperature effects in stationary phase, due to the reduction of temperature dependence caused by nutrient limitation (O’Connor *et al.,* 2009; Marañón *et al.,* 2018; Qu *et al.,* 2019). However, our data did not clearly show this decrease of temperature dependence despite nitrate concentrations being below the detection limit during the stationary phase in all species. This could reflect the difference between conditions of transient nutrient starvation during the stationary phase in batch cultures and the chronic nutrient limitation status experienced by cells in chemostats (Marañón *et al.,* 2018) as well as in the oligotrophic subtropical gyres (Fernández-González *et al.,* 2022) and during long-term experiments over evolutionary time scales (Aranguren-Gassis *et al.,* 2019). Moreover, some species (e.g. diatoms) are able to maintain their photosynthetic carbon fixation during a certain time despite the lack of nitrogen in the extracellular medium, due to their ability to store nutrients intracellularly (López-Sandoval *et al.,* 2014). Comparatively few studies have addressed nutrient starvation in the stationary phase, which is characterized by high cell density, more dead cells and potential light shading. These conditions mimic the end of a bloom in nature, where populations are not always growing exponentially but are often subject, at least in coastal regions, to bloom-and-bust dynamics. The fact that we conducted our measurements during both phases strengthens the conclusion that fluctuations tended to stimulate photosynthesis compared to constant temperature conditions.

## CONCLUSION

We have shown that the phytoplankton responses to the combined effect of thermal fluctuations and warming under nutrient replete and limited conditions are highly trait- and species-specific. Fluctuating temperatures tended to cause a decrease of POC-growth rates, which was accentuated under warming conditions. By contrast, fluctuation stimulated photosynthesis both in the exponential growth and the stationary phase. Given that the responses of the physiological traits differed between species and play a key role in determining how changes in temperature may influence metabolic balance, trophic interactions or community and size structure in marine ecosystems, it becomes necessary to apply the experimental approach used here to investigate the effect of thermal fluctuations in natural communities.

## Supplementary Material

Suppl_table1_fbaf021

## Data Availability

Data supporting the findings of this study are available from the corresponding author upon reasonable request.
