# Supplementary material for: Impact of thermal fluctuations on phytoplankton: an experimental multi-trait analysis across species
Source: J Plankton Res. 2025 May 21;47(3):fbaf021. doi: 10.1093/plankt/fbaf021 (PMC12094783; doi:10.1093/plankt/fbaf021)
Supplement: Suppl_table1_fbaf021 [file suppl_table1_fbaf021.docx]

**Supplementary material**

|  | **Exponential phase** | | | **Stationary phase** | | |
| --- | --- | --- | --- | --- | --- | --- |
|  | **PC1** | **PC2** | **PC3** | **PC1** | **PC2** | **PC3** |
| **Eigenvalue** | 3.49 | 1.80 | 1.29 | 1.90 | 1.57 | 1.35 |
| **Explained variance (%)** | 43.65 | 22.49 | 16.07 | 31.67 | 26.19 | 22.58 |
| **Cumulative proportions** | 43.65 | 66.14 | 82.21 | 31.67 | 57.86 | 80.44 |
| **Trait contributions** |  |  |  |  |  |  |
| **POC-based growth rate** | 0.03 | 43.62 | 0.90 | - | - | - |
| **Chl *a*-based growth rate** | 19.49 | 3.32 | 0.06 | - | - | - |
| **C:N** | 15.97 | 5.51 | 12.67 | 37.01 | 0.02 | 14.84 |
| **C:Chl *a*** | 0.11 | 22.98 | 23.42 | 3.41 | 20.96 | 4.87 |
| **Photosynthesis** | 2.10 | 5.56 | 59.10 | 11.36 | 37.87 | 6.66 |
| **Respiration** | 22.86 | 2.26 | 0.01 | 41.95 | 0.04 | 7.28 |
| **Fv/Fm** | 22.41 | 1.95 | 1.70 | 4.89 | 41.05 | 1.45 |
| **rETR_max_** | 17.04 | 14.79 | 2.13 | 1.38 | 0.06 | 64.89 |

Table S1. Summary of the principal component analysis (PCA) for the eight and six traits measured during the exponential growth and stationary phases, respectively. POC, C:Chl *a*, Fv/Fm and rETR_max_ refer to particulate organic carbon, carbon-to-chlorophyll *a* ratio, maximum photochemical yield of photosystem II and relative maximum electron transport rate, respectively.
